# Beryllium und seine anorganischen Verbindungen – Addendum: Reevaluierung des BAR: Beurteilungswerte in biologischem Material

**DOI:** 10.34865/bb744041d9_4ad

**Published:** 2024-12-20

**Authors:** Julia Hiller, Wobbeke Weistenhöfer, Hans Drexler, Andrea Hartwig, MAK Commission

**Affiliations:** 1 Friedrich-Alexander-Universität Erlangen-Nürnberg. Institut und Poliklinik für Arbeits-, Sozial- und Umweltmedizin Henkestraße 9–11 91054 Erlangen Deutschland; 2 Institut für Angewandte Biowissenschaften. Abteilung Lebensmittelchemie und Toxikologie. Karlsruher Institut für Technologie (KIT) Adenauerring 20a, Geb. 50.41 76131 Karlsruhe Deutschland; 3 Ständige Senatskommission zur Prüfung gesundheitsschädlicher Arbeitsstoffe. Deutsche Forschungsgemeinschaft, Kennedyallee 40, 53175 Bonn, Deutschland. Weitere Informationen: Ständige Senatskommission zur Prüfung gesundheitsschädlicher Arbeitsstoffe | DFG

**Keywords:** Beryllium, Biologischer Arbeitsstoff-Referenzwert, BAR, Expositionsäquivalente für krebserzeugende Arbeitsstoffe, EKA, Urin, 7440-41-7, urine, 7440-41-7

## Abstract

The German Senate Commission for the Investigation of Health Hazards of Chemical Compounds in the Work Area (MAK Commission) summarised and re-evaluated the data for beryllium and its inorganic compounds regarding the recommended sampling times for biomonitoring, the derivation of exposure equivalents for carcinogenic substances (EKA) and the biological reference value (BAR) in urine. Only one new study reports data for beryllium in air and urine, but for the same beryllium concentration in air, the corresponding concentration in urine was lower than that given in two older publications. Considering the inconsistent data and questions on the validity of older analytical methods, the data base is not sufficient to derive EKA. The BAR was re-evaluated and a BAR of 0.02 µg/l urine was established based on the data for background exposure. Sampling should be conducted after a steady state has been reached. The data base is not sufficient for the evaluation of a BAR in blood.

**Table:** 

**BAR (2023)**	**0,02 µg Beryllium/l Urin**
	Probenahmezeitpunkt: keine Beschränkung im Fließgleichgewicht; aufgrund der langen Halbwertszeit kann es nach (Wieder-)Aufnahme der Tätigkeit mehrere Wochen dauern, bis sich ein Fließgleichgewicht einstellt
**EKA**	**nicht festgelegt**
	
**MAK-Wert (2003)**	**–**
Hautresorption	–
Krebserzeugende Wirkung (2003)	Kategorie 1
Sensibilisierende Wirkung (2002)	Sah

## Reevaluierung

Beryllium und seine anorganischen Verbindungen wurden 2002 und 2009 (re)evaluiert, wobei die vorliegenden Daten nicht ausreichend waren, um Expositionsäquivalente für krebserzeugende Arbeitsstoffe (EKA) abzuleiten (Schaller 2003, 2010). Im Jahr 2009 wurde ein Biologischer Arbeitsstoff-Referenzwert (BAR) von 0,05 µg/l Urin abgeleitet (Schaller 2010). Im vorliegenden Addendum wird die Möglichkeit der Ableitung von EKA geprüft. Weiterhin werden der BAR sowie der Probenahmezeitpunkt reevaluiert. 

Bei der Bewertung von Messdaten zu Beryllium ist zu berücksichtigen, dass in älteren Arbeiten (vor dem Jahr 2000) häufig analytische Methoden verwendet wurden, welche nach neueren Erkenntnissen hinsichtlich Sensitivität und Spezifität nicht ausreichend sind, um zuverlässig niedrige Berylliumkonzentrationen zu messen (vgl. Schaller 2003). Für ein verlässliches Biomonitoring von Beryllium wurde daher ein Einsatz von modernen Analysemethoden wie Massenspektrometrie mit induktiv gekoppeltem Plasma (ICP-MS) oder optimierte Atomabsorptionsspektrometrie mit elektrothermischer Aufheizung (GF-AAS-Techniken) empfohlen (Schaller 2003, 2010). Ältere Arbeiten, die diese Kriterien nicht erfüllen, sind daher hinsichtlich ihrer Aussagekraft nur eingeschränkt für die Bewertung heranziehbar.

## Metabolismus und Toxikokinetik 

### Humandaten

Bei beruflichen Belastungen mit Beryllium überwiegt der inhalative Aufnahmeweg. Daten über den Umfang einer möglichen Ablagerung von Beryllium in der Lunge bzw. einer inhalativen Bioverfügbarkeit lagen bei den letzten umfassenden Stoffbewertungen nicht vor (vgl. Greim 2003; Schaller 2003). In zwei älteren Arbeiten (Stiefel et al. 1980; Zorn et al. 1986) wurden jedoch Biomonitoring-Daten nach akzidentellen inhalativen Belastungen beschrieben. Neben der als fraglich zu bewertenden analytischen Qualität (u. a. Messwerte bei nicht-exponierten Personen deutlich höher als nach heutigem Stand zu erwarten; ca. 1 µg/l Urin) sind bei beiden Arbeiten die Daten teils nicht nachvollziehbar. Stiefel et al. (1980) berichteten über akzidentell gegen BeCl_2_ exponierte Beschäftigte eines Labors (n = 8), bei denen es bei Luftkonzentrationen von bis zu ca. 8 ng/m^3^ zu einem zeitgleichen Anstieg der Berylliumkonzentration im Urin auf etwa das 4-Fache des Ausgangswertes und anschließendem Rückgang über ca. 10–15 Tage gekommen war. Zorn et al. (1986) berichteten über eine unfallartige Exposition gegen Berylliumstaub bei 25 Personen. Einen Tag nach der Exposition betrug die mittlere Berylliumkonzentration im Serum 3,5 ± 0,47 ppb (= µg/l) und sieben Tage nach der Exposition 2,4 ± 0,3 µg/l. Mit Ausnahme der am höchsten belasteten Person lagen alle weiteren Messungen nach 2 bis 8 Wochen im Referenzbereich (hier angenommen bis 1 µg/l). Für die am stärksten belastete Person wurden Serumkonzentrationen von 5,2 µg/l, 3,6 µg/l und ca. 2 µg/l einen bzw. sieben Tage sowie 6 Monate nach der Exposition angegeben. Von den Autoren wurde aufgrund dieser Daten eine biologische Halbwertszeit beim Menschen von ca. 2 bis 8 Wochen nach einer einmaligen inhalativen Exposition abgeschätzt. 

Seitdem wurden zwei weitere Arbeiten mit humanen Eliminationsdaten nach Unfallereignissen veröffentlicht. Aviv et al. (2018) berichteten über eine kurzzeitige inhalative Exposition von Beschäftigten gegen das Radioisotop ^7^Be. Bei einem Freiwilligen wurde mittels Aktivitätsmonitoring die Lungenbelastung mit ^7^Be über 108 Tage nachverfolgt und zusätzlich die Aktivität in vier Spoturinproben aus den ersten drei Tagen gemessen. Für die Lunge wurde hieraus eine zweiphasige Elimination mit einer schnellen ersten Phase mit einer zerfallskorrigierten Halbwertszeit zwischen 0,4 und 1 Tag, sowie eine zweite langsamere Elimination mit einer Halbwertszeit von ca. 109 Tagen ermittelt. Eine geringe Aktivität von ^7^Be war auch in den Urinproben nachweisbar, so dass anzunehmen ist, dass zumindest ein kleiner Anteil der inhalativ aufgenommenen Menge systemisch verfügbar wurde. Die höchste Aktivitätskonzentration im Urin zeigte sich dabei einen Tag nach der Exposition (0,2 Tage nach der Exposition lag die Aktivität im Urin bei 7,5 Bq/l; nach einem Tag bei 10,5 Bq/l, nach 1,9 Tagen bei 7,5 Bq/l und nach 3,0 Tagen bei 1,3 Bq/l). Die rasche anfängliche Phase der Eliminierung der Aktivität aus der Lunge könnte möglicherweise auf eine erhebliche mukoziliäre Clearance zurückzuführen sein. Von Hiller et al. (2023) wurde eine Berylliumexposition über direkten Gewebe- und Bluteintrag durch ein Explosionstrauma berichtet (Explosion eines mit 2,0 g Beryllium gefüllten Glaskolbens in der Hand eines Labormitarbeiters mit nachfolgend ausgedehnter Weichteilschädigung). Aus dem mehrjährigen medizinischen Follow-up inklusive Biomonitoring im Urin (beginnend an Tag 2 nach Exposition) und Blut (beginnend an Tag 147) wurde eine zweiphasige Eliminationskinetik im Urin mit Halbwertszeiten von 117 bzw. 666 Tagen und eine einphasige Eliminationskinetik im Blut mit einer Halbwertszeit von 103 Tagen ermittelt. Die maximalen Berylliumkonzentrationen lagen im Urin bei 4,48 µg/l an Tag 2 und im Vollblut bei 1,41 µg/l an Tag 147. Einschränkend ist hier zu berücksichtigen, dass die Belastung im Blut erst mit einer Verzögerung von ca. 5 Monaten erfasst wurde. Zudem ist auch eine fortgesetzte subakute Berylliumbelastung über in das Gewebe eingesprengte Partikel nicht auszuschließen. Es wurde jedoch von einer im Vordergrund stehenden akuten systemischen Belastung ausgegangen. 

Aus neueren Feldstudien (mit ausreichend sensitiver Analytik) liegen zudem weitere Daten vor, die Anhaltspunkte zu zeitlichen Aspekten eines Belastungsmonitorings in beruflich exponierten Kollektiven liefern können. Wegner et al. (2000) und Bounaurio et al. (2021) beschrieben jeweils (statistisch nicht signifikant) höhere Berylliumkonzentrationen im Urin in Vorschichtproben im Vergleich zu Nachschichtproben. Morton et al. (2011) fanden hingegen eine um 47 % statistisch signifikant höhere Berylliumausscheidung in Urinproben, die am Ende der Woche gewonnen wurden, im Vergleich zu korrespondierenden Proben vom Wochenanfang. Eine mehrjährige Studie von Devoy et al. (2019) mit Angaben zu Berylliumkonzentrationen in der Luft und im Urin von exponierten Beschäftigten aus fünf verschiedenen französischen Betrieben analysierte mögliche Zusammenhänge zwischen äußeren und inneren Belastungen (ausführlichere Darstellung siehe Abschnitt Beziehung zwischen äußerer und innerer Belastung).

### Daten aus Tierversuchen

Vorliegende **tierexperimentelle Daten aus nicht-inhalativer Exposition** (intravenös, intramuskulär, subkutan, intraperitoneal) sind überwiegend nicht geeignet, um toxikokinetische Daten zur Elimination abzuleiten. Oft werden nur die Verteilung auf verschiedene Organe bzw. Gewebe (Hard et al. 1977; Lindenschmidt et al. 1986) oder Daten über die Ganzkörperretention (Furchner et al. 1973; Sakaguchi et al. 1993) beschrieben. 

T**ierexperimentelle Daten nach inhalativer Exposition** zeigen eine lange zweiphasige pulmonale Clearance in Abhängigkeit von der untersuchten Berylliumverbindung, Menge und Tierart (Benson et al. 2000; Finch et al. 1990; Greim 2003; Haley et al. 1989, 1990; Rhoads und Sanders 1985; Sanders et al. 1975; Schaller 2003). 

Es liegen auch **tierexperimentelle Daten zu Berylliumkonzentrationen im Blut oder Urin nach inhalativen Expositionen** vor. Von Stiefel et al. (1980) wurde eine erste Elimination von inhalativ aufgenommenem Beryllium (^4^Be-Salz-Aerosole) im Urin von Meerschweinchen nach 2 Stunden beschrieben. Das Eliminationsmaximum lag 10 Stunden nach Ende der 8- bis 16-stündigen Exposition. Die Konzentrationen im Urin erreichten innerhalb von 5 Tagen nach der Maximalkonzentration wieder „normale Werte“. Der Anstieg der Berylliumkonzentration im Serum verlief parallel zu dem Anstieg im Urin, der Zeitverlauf der Elimination im Urin und Serum ist daher sehr ähnlich. Finch et al. (1990) ermittelten für eine kurzzeitige (5–42 Minuten) nasale Exposition von Hunden mit BeO in Abhängigkeit vom Herstellungsverfahren eine Halbwertszeit für die Elimination im Urin von 103 bzw. 58 Tagen. Bei der Bewertung dieser beiden Arbeiten muss jedoch die geringe Sensitivität und Spezifität früherer Analysemethoden berücksichtigt werden. Von Muller et al. (2010) wurde eine subakute nasale Inhalation (250 µg/m^3^ für 6 h/Tag, 5 Tage/Woche, 3 Wochen) mit verschiedenen Berylliumverbindungen (metallisches Be, BeO, BeAl) an Mäusen untersucht und die Urinkonzentration nach der ersten, zweiten und dritten Woche der Exposition sowie eine Woche nach Ende der Exposition gemessen. Der Konzentrationsverlauf im Urin unter Exposition sowie die Höhe des Rückgangs nach Expositionsende waren abhängig von der Verbindung. 

## Belastung und Beanspruchung 

### Beziehung zwischen äußerer und innerer Belastung 

Aufgrund der Einstufung von Beryllium als Kanzerogen der Kategorie 1 wurde im Jahr 2003 die Ableitung von EKA geprüft, wobei die vorliegenden Daten als nicht ausreichend bewertet wurden (Schaller 2003).

Für eine Neubewertung der inneren und äußeren Exposition werden folgende neuere Publikationen herangezogen: 

Morton et al. (2011) untersuchten 167 Beschäftigte aus der Aluminium-Produktion und dem Recycling. Die mittels ICP-MS bestimmten Berylliumkonzentrationen im Urin lagen in einem Bereich von unter der Bestimmungsgrenze bis 0,178 µg/l Urin, bei einem Mittel von 0,0195 µg/l Urin. Für 135 exponierte Beschäftigte lagen Urinproben vom Anfang und Ende der Wochen vor. Die exponierten Beschäftigten hatten am Ende der Woche 47 % höhere Berylliumwerte im Urin als am Wochenbeginn. Von Bena et al. (2020) wurde ein mehrjähriges Verlaufsmonitoring von Beschäftigten einer Abfallverbrennungsanlage in Italien vor bzw. ein und drei Jahre nach Inbetriebnahme der Anlage beschrieben. Die Berylliumkonzentration im Urin von 26 in der Verbrennungsanlage Beschäftigten, für die ein vollständiger Verlauf verfügbar war, war über die Zeit eher rückläufig (vor Inbetriebnahme: Median: 0,19 µg Beryllium/l Urin; 95. Perzentil: 0,29 µg/l; nach 3 Jahren: Median: 0,09 µg Beryllium/l Urin; 95. Perzentil: 0,13 µg/l). Eine Kontrollgruppe von 9 administrativ Beschäftigten zeigte einen vergleichbaren Trend und ähnliche Werte (vor Inbetriebnahme: Median: 0,10 µg Beryllium/l Urin; 95. Perzentil: 0,37 µg/l; nach 3 Jahren: Median: 0,08 µg Beryllium/l Urin; 95. Perzentil: 0,16 µg/l). Insgesamt lagen die Urinbelastungen in diesem Kollektiv – insbesondere vor Inbetriebnahme der Anlage – eher höher, als in beruflich nicht exponierten Kollektiven zu erwarten wäre (im Vergleich zum damaligen BAR von 0,05 µg/l Urin). Bounaurio et al. (2021) untersuchten den Urin von 40 Metallarbeitern/Schweißern u. a. in Bezug auf die Berylliumkonzentration in Vor- und Nachschichtproben. Hierbei waren die Berylliumkonzentrationen im Urin in den Vorschichtproben (Mittelwert: 0,019 µg Beryllium/g Kreatinin; 95. Perzentil: 0,039 µg/g Kreatinin) höher als in den Nachschichtproben (Mittelwert: 0,014 µg Beryllium/g Kreatinin; 95. Perzentil: 0,020 µg/g Kreatinin). Dieser Unterschied war jedoch nicht statistisch signifikant. 

Von Hulo et al. (2016) wurde für 30 gegen Beryllium exponierte Beschäftigte (und 21 nicht-exponierte Kontrollpersonen) aus einem Aluminiumproduktionsbetrieb kein statistisch signifikanter Zusammenhang zwischen der kumulierten Berylliumexposition und der renalen Berylliumausscheidung beobachtet. Die beschriebene statistisch signifikante Korrelation des kumulativen Beryllium-Expositions-Index mit der Berylliumkonzentration im exhaliertem Atemkondensat spiegelt nicht notwendigerweise eine innere Exposition wider. 

In zwei der drei neueren Feldstudien mit simultaner Untersuchung von Luft- und Urinproben konnten trotz der eingesetzten ICP-MS-Technik für die Analytik der Luftproben und GF-AAS-Methoden für die Analytik der Urinproben bei fraglicher beruflicher Exposition keine Belastungen quantifiziert werden (Gerding et al. 2021; Godderis et al. 2005). In der Studie von Devoy et al. (2019) wurden über einen Zeitraum von 5 Jahren luftgetragene Berylliumexpositionen und dadurch verursachte Urinbelastungen bei insgesamt 75 Beschäftigten aus fünf verschiedenen französischen Betrieben untersucht und ein Korrelationsmodell abgeleitet. Aufgrund optimierter ICP-MS-Analysemethoden nahm die Sensitivität im Studienverlauf zu. Luftproben wurden über einen 8-Stunden-Arbeitstag mittels personengetragener Luftpumpen gewonnen (Flussrate 2 l/min), die Bestimmungsgrenzen lagen je nach Betrieb zwischen 0,11 und 100 ng/Filter. Parallel zu den Luftmessungen erfolgten Urinsammlungen über mindestens fünf Tage, teils mit mehreren Proben pro Tag und bei einigen Beschäftigten auch zusätzlich an zwei expositionsfreien Tagen. 2600 Urinproben mit akzeptabler Urinverdünnung konnten in die Auswertung eingeschlossen werden. Die Bestimmungsgrenzen für Beryllium im Urin lagen bei 0,1 µg/l im Betrieb A (Gießerei für Kupfer-Beryllium-Verbindungen (CuBe)), bei 0,02 µg/l im Betrieb B (Aluminium-Beryllium (AIBe) Maschinenbauunternehmen) und C (Unternehmen für die Bearbeitung von CuBe Teilen) und bei 0,002 g/l in Betrieb D (Aluminiumschmelzerei) und E (Aluminiumgießerei). Da in den Betrieben B und C Beryllium in allen Urinproben unterhalb der Bestimmungsgrenze (BG) lag und nur in 3 bzw. 12 % der Luftfilter bestimmbar (BG–2,5 ng/Filter) war, wurden die Daten aus diesen beiden Betrieben nicht in die weiterführenden Analysen aufgenommen. Aus den Betrieben A, D und E lagen von 41 Beschäftigten 187 Luftmessungen (Bereich: < BG–7,58 µg Beryllium/m^3^; geometrisches Mittel: 0,035 µg Beryllium/m^3^) und 1350 Urinproben (Bereich: < BG–0,35 µg Beryllium/g Kreatinin; geometrisches Mittel: 0,0047 µg Beryllium/g Kreatinin) vor. Für die Beziehung zwischen der jeweiligen mittleren externen und inneren Belastung ergab sich ein statistisch signifikanter Zusammenhang. Eine Konzentration von 1 µg Beryllium/m^3^ korrespondierte dabei mit einer geschätzten Berylliumausscheidung von 0,047 µg/g Kreatinin. Wenngleich ein Gesamtmodell zeigte, dass die Arbeiter-spezifische mittlere Berylliumausscheidung im Urin statistisch signifikant mit der jeweiligen mittleren Berylliumexposition in der Luft anstieg, ergaben weiterführende kinetische Analysen der individuellen Exkretionsprofile keinen unmittelbaren Bezug zur Konzentration in der Luft. Insbesondere zeigte sich kein Exkretionspeak in den Stunden unmittelbar nach Exposition und die Urinausscheidung sank auch nicht während zweitägiger expositionsfreier Phasen. Die Autoren schlussfolgerten daraus, dass die gemessenen Berylliumkonzentrationen im Urin nicht die jüngsten äußeren Expositionen widerspiegeln und die Halbwertszeit der renalen Elimination bei mehr als 48 Stunden liegt. Der abgeleitete Zusammenhang zwischen Urin- und Luftkonzentrationen reflektiert daher eine chronische Belastung nach Einstellung eines Fließgleichgewichts. 

## Reevaluierung der EKA

Aktuell liegen lediglich drei Arbeiten mit Luftmessungen und korrespondierenden Urindaten vor (Apostoli und Schaller 2001; Devoy et al. 2019; Zorn et al. 1988), wobei sich ein sehr uneinheitliches Bild bezüglich der korrespondierenden Luft- und Urinkonzentrationen zeigt (vgl. Tabelle 1). wurden in älteren Studien Analysemethoden mit vermutlich nicht ausreichender Sensitivität und Spezifität verwendet; insbesondere die Validität der von Zorn et al. (1988) beschriebenen Beziehung wurde als sehr gering eingestuft (Schaller 2003).

**Tab. 1:** Berylliumkonzentrationen in Luft und Urin bei beruflich Exponierten

**Beryllium**		**Literatur**
**Luft** **[µg/m^3^]**	**Urin** **[µg/l]**		
0,2 (2)	0,7 (7)	Zorn et al. 1988
0,2	0,12–0,15	Apostoli und Schaller 2001
1 *bezogen auf 0,2*	0,047 µg/g Kreatinin ≙ 0,056 µg/l^a)^ *0,011*	Devoy et al. 2019

^a)^ unter Ansatz des Umrechnungsfaktors von 1,2 g Kreatinin/l für gemischte Kollektive bei Umrechnung von Kreatininbezug auf Volumenbezug    (Bader et al. 2020)

Aufgrund der inkonsistenten Datenlage lassen sich daher weiterhin keine ausreichend begründeten EKA ableiten. 

## Hintergrundbelastung

Die Bestimmung der Hintergrundbelastung ist nur mit sensitiven Analysemethoden (Bestimmungsgrenzen im Bereich von 0,01 µg Beryllium/l) möglich.

Seit 2000 wurden folgende Daten zur Hintergrundbelastung von Erwachsenen mit Beryllium in Urin oder Blut publiziert (Tabelle 2):

**Tab. 2:** Konzentrationen von Beryllium in Urin bzw. Blut, Plasma oder Serum bei Personen der Allgemeinbevölkerung und in Kollektiven ohne berufliche Berylliumexposition (Studien ab 2000; ICP-MS; Werte unterhalb der Bestimmungsgrenze (BG) kursiv (bei Bedarf extrapoliert vom 3-Fachen der Nachweisgrenze (NWG)))

**Land**	**Kollektiv, Alter**	**Beryllium [µg/l]**			**Literatur**
		**NWG/BG**	**Urin**	**Blut, Plasma oder Serum**	
Deutschland	30 Beschäftigte ohne Metall-Exposition Alter: MW 38,3 ± 11,5 Jahre	NWG: 0,03 BG: k. A.	*Median: < 0,03* P95: k. A. Bereich: k. A.	–	Apostoli und Schaller 2001
Deutschland	63 Personen der Allgemeinbevölkerung Alter: ≥ 18 Jahre	NWG: 0,004 BG: 0,014	*GM: 0,007* P95: 0,020 Bereich: *BG*–0,028	–	Heitland und Köster 2004
Frankreich	100 gesunde Personen	NWG: 0,015 BG: 0,05	*Median: 0,01* *P95: 0,042* Bereich (P5–P95): *0,008–0,042*	–	Goullé et al. 2005
		NWG: 0,03 BG: 0,10	–	Plasma *Median: 0,015* P95: 0,103 Bereich (P5–P95): *0,015*–0,103 Vollblut BG > 0,1	
Deutschland	87 Personen der Allgemeinbevölkerung 40 ♂, 43 ♀ Alter: 18–65 Jahre	NWG: k. A. BG: 0,009	*GM: < BG* *P95: < BG* *Bereich: < BG*	–	Heitland und Köster 2006 a
Deutschland	130 Personen der Allgemeinbevölkerung 50 ♂, 80 ♀ Alter: 18–70 Jahre	NWG: k. A. BG: 0,008	–	Vollblut *GM: < 0,008* P95: 0,015 Bereich: *< 0,008–*0,04	Heitland und Köster 2006 b
Tokio	78 gesunde Schwangere (9.–40. SSW) Alter: 22–42 Jahre, MW 32,1 Jahre	NWG: 0,03 µg/g Krea (= 0,024 µg/l) BG: k. A.	*GM: 0,031 µg/g Krea* P95: k. A. Bereich: *< 0,03*–0,685 µg/g Krea	–	Shirai et al. 2010
Italien	104 (A; Umbrien) bzw. 106 (B; Calabrien) Personen der Allgemeinbevölkerung	NWG: 0,022 BG: k. A.	–	Serum *GM: 0,06 (A) / 0,05 (B)* P95: 0,09 (A + B) Bereich: *0,02*–0,13 (A) bzw. *0,02*–0,14 (B)	Bocca et al. 2010
UK	62 beruflich nicht-exponierte Personen	NWG: 0,002 BG: 0,006	GM: 0,0095 (P90: 0,020) P95: 0,031^a)^ Bereich: *< NWG*–0,044	–	Morton et al. 2011
UK	111 Patienten mit Nierensteinen 77 ♂, 34 ♀ Alter: 21–85 Jahre, Median 51,5 Jahre	NWG: 1,1 nmol/l (= 0,0099 µg/l) BG: k. A. (94,6 % < NWG)	*Median: < NWG* P95: k. A. *Bereich (P2,5–P97,5): < NWG–2,7 nmol/24 h (0,0243 µg/24 h)*	–	Sieniawska et al. 2012
Belgien	1001 Personen der Allgemeinbevölkerung 460 ♂, 541 ♀ Alter: 18–80 Jahre, MW 40,1 Jahre	NWG: 0,007 BG: 0,022	*Median: < NWG* *P97,5: < NWG* Bereich: k. A.	–	Hoet et al. 2013
Frankreich	nicht gegen Beryllium exponierte Beschäftigte aus Betrieben der Aluminiumproduktion bzw. optoelektronischen Industrie k. A. zu Anzahl, Alter oder Geschlecht	NWG: 0,0006 BG: 0,0025	*GM: k. A.* *P95: k. A.* *Bereich: < NWG*	–	Devoy et al. 2013
UK	132 Personen der Allgemeinbevölkerung 82 ♂, 50 ♀ Alter: 18–66 Jahre	NWG: k. A. BG: 0,0006	280 Proben (teils mehrere Proben pro Person) Median: 0,0052 P95: 0,0116 Bereich: k. A.	–	Morton et al. 2014
Spanien	21 nicht gegen Beryllium exponierte Beschäftigte aus einer Aluminiumproduktionsanlage 10 ♂, 11 ♀ Alter: 36–49 Jahre, Median 44 Jahre	NWG: k. A. BG: 0,0064	MW: 0,0152 µg/g Krea P95: k. A. Bereich: k. A. (90 % > BG)	–	Hulo et al. 2016
Frankreich	2000 Personen der Allgemeinbevölkerung^b)^ 982 ♂, 1018 ♀ Alter: 20–59 Jahre				Nisse et al. 2017
	1910 Personen; 942 ♂, 968 ♀	NWG (Urin): 0,0006 BG: k. A.	AM: 0,04 GM: 0,004 P95: 0,15 Bereich (P10–P95): < NWG–0,15 (41,6 % < NWG)		
	1992 Personen; 976 ♂, 1016 ♀	NWG (Blut): 0,0004 BG: k. A.		Vollblut AM: 0,02 GM: 0,003 P95: 0,09 Bereich (P10–P95): < NWG–0,09 (42,7 % < NWG)	
USA	390 Schwangere (2.–3. Trimenon) Alter: Median 32,2 Jahre	NWG: 0,04 BG: k. A.	*GM: 0,02* *P95: 0,11* Bereich: k. A. (91,3 % < NWG)	–	Kim et al. 2018
Spanien	21 ♂, Leistungssportler (A), 26 ♂, Studenten („sedentary“; B) Alter: MW ca. 22–23 Jahre	NWG: 0,034 BG: k. A.	MW: 0,822 (A) bzw. 0,116 (B) P95: k. A. Bereich: k. A.	Serum *MW: 0,09 (A)* *bzw. 0,04 (B)* P95: k. A. Bereich: k. A.	Maynar et al. 2018
USA	1335 ♀ aus SWAN-Studie^c)^ Alter: 45–56 Jahre, MW 49,4 Jahre	NWG: 0,04 BG: k. A.	*GM: nicht bestimmt* (83,8 % < NWG) *P95: 0,08* Bereich (P5-P95): *< NWG–0,08*	–	Wang et al. 2019
Italien	35 Beschäftigte einer Müllverbrennungsanlage: (A) 9 ♂ administrativ und (B) 26 ♂ in der Anlage beschäftigt, bevor die Anlage in Betrieb genommen wurde Alter: MW 42,8 Jahre (A), 38,5 Jahre (B)	NWG: 0,04 BG: k. A.	Median: *0,10 (A)* bzw. 0,19 (B) P95: 0,37 (A) bzw. 0,29 (B) Bereich: k. A.	–	Bena et al. 2020
Belgien	380 Personen der Allgemeinbevölkerung 178 ♂, 202 ♀ Alter: 18–70 Jahre, MW 35,4 Jahre	NWG: 0,03 BG: 0,08	–	Vollblut *Median: < NWG* *P97,5: < NWG* Bereich: k. A.	Hoet et al. 2021
Deutschland	102 Personen der Allgemeinbevölkerung 38 ♂, 64 ♀ Alter: 19–66 Jahre	NWG: k. A. BG: 0,02 (Urin und Vollblut)	*GM: < 0,02* *P95: < 0,02* *Bereich: < 0,02*	Vollblut *GM: < 0,02* *P95: < 0,02* *Bereich: < 0,02*	Heitland und Köster 2021
		NWG: k. A. BG: 0,004 (Serum)	–	Serum *GM: < 0,004* *P95: < 0,004* *Bereich: < 0,004 *	
Deutschland	20 Büro-Beschäftigte aus Elektromüllrecycling-Betrieben 15 ♂, 5 ♀ Alter: 27–59 Jahre, MW 50,6 Jahre	NWG: 0,03 BG: 0,09	*GM: 0,02* *P95: 0,05* *Bereich: 0,02–0,05*	–	Gerding et al. 2021
Italien	13 ♂ unbelastete Beschäftigte eines metallverarbeitenden Betriebs Alter: 20–59 Jahre, MW 44,9 Jahre	NWG: 0,03 BG: 0,09	*MW: 0,01 µg/g Krea* *P95: 0,015 µg/g Krea* *Bereich (P5–P95): 0,007–0,015 µg/g Krea^d)^*	–	Buonaurio et al. 2021
Deutschland	77 Personen der Allgemeinbevölkerung 34 ♂, 43 ♀ Alter: 19–78 Jahre, MW 45,8 Jahre	NWG: k. A. BG: 0,003	*GM: k. A.* *P90: < BG* *P95: < BG^e)^* Bereich:*< BG*–0,005 (98 % < BG)	–	Schmied et al. 2021
China	63 Bürobeschäftigte ohne Metallexposition 46 ♂, 17 ♀ Alter: MW 39,3 ± 11,4 Jahre	NWG: 0,007 BG: k. A.	*MW: 0,0178* P95: 0,0245 Bereich: *0,0133–*0,0299	Serum MW: 0,0264 P95: 0,0486 Bereich: *0,0038*–0,0584	Liu et al. 2021
Norwegen	757 gesunde Personen der Allgemeinbevölkerung^f)^ 375 ♂, 382 ♀ Alter: 49–66 Jahre, MW 57,4 Jahre	NWG: 0,02 BG: k. A.	–	Vollblut MW: 0,035 *Median: < 0,02* P90: 0,088 Bereich: *< 0,020*–0,359^h)^	Syversen et al. 2021
Norwegen	1011 Personen der Allgemeinbevölkerung^g)^ 506 ♂, 505 ♀ Alter: 20–91 Jahre, MW 50,0 ± 17,6 Jahre	NWG: 0,0096 BG: k. A.	–	Vollblut *GM: < 0,0096* *P95: 0,0122* *Bereich:* *< 0,0096–0,0260*^h)^	Simić et al. 2022

^a)^ Morton 2023

^b)^ früher stark industriell belastetes Gebiet

^c)^ longitudinale Verlaufsstudie an Frauen in der Menopause

^d)^ Werte kalkuliert mit NWG/2 => nicht sinnvoll verwertbar, da vermutlich unter BG; k. A. zu Kreatiningehalten im Urin

^e)^ Schmied 2023

^f)^ Teilkollektiv 1 aus HUNT3-Studie: The Nord-Trøndelag Health Survey von 2006–2008; initial selektioniert für Studie zur Neurobildgebung

^g)^ Teilkollektiv 2 aus HUNT3-Studie, nicht identisch mit den Personen von Syversen et al. (2021)

^h)^ Unterschiede zwischen den Ergebnissen von Syversen et al. (2021) und Simić et al. (2022) am ehesten durch verlässlichere Analyse der Studie von Simić bedingt (Syversen 2023)

AM: arithmetischer Mittelwert; GM: geometrischer Mittelwert; k. A.: keine Angabe; Krea: Kreatinin; MW: Mittelwert; P2,5, P5, P10, P90, P95, P97,5: 2,5., 5., 10., 90., 95., 97,5. Perzentil; SSW: Schwangerschaftswoche

## Reevaluierung des BAR

Zu Berylliumkonzentrationen **im Blut und Serum** liegen vergleichsweise wenige Studien vor (Heitland und Köster 2006 b; Hoet et al. 2021; Syversen et al. 2021), so dass kein BAR für Beryllium im Blut evaluiert wird.

Hinsichtlich der Reevaluierung des BAR **im Urin** wurden bevorzugt Daten aus der deutschen Bevölkerung und im Weiteren aus dem europäischen Raum herangezogen. Dabei wurden nur Arbeiten berücksichtigt, in denen die Bestimmungsgrenze ≤ 0,05 µg/l (entsprechend dem bisherigen BAR) bzw. die Nachweisgrenze ≤ 0,02 µg/l war. Tabelle 3 zeigt eine Übersicht aller Studien mit ausreichender analytischer Sensitivität und Spezifität und für die Ableitung eines BAR repräsentativen Kollektiven, in denen ein 95. Perzentil der Berylliumkonzentration im Urin angegeben wurde. 

**Tab. 3:** Bewertungsrelevante Studien zur Hintergrundbelastung der Allgemeinbevölkerung mit Beryllium im Urin

**Anzahl der Personen**	**95. Perzentil Beryllium [µg/l Urin]**	**Literatur**
Europäische Studien		
64	0,02	Heitland und Köster 2004
62	0,031^a)^	Morton et al. 2011
132	0,012	Morton et al. 2014
1910	0,15	Nisse et al. 2017
Europäische Studien mit 95. Perzentil < BG		
100	< BG (0,05) *0,042*^b)^	Goullé et al. 2005
87	< BG (0,009)	Heitland und Köster 2006 a
111	≈ NWG (0,0099) (94,6 % < NWG)	Sieniawska et al. 2012
1001	< NWG (0,007)	Hoet et al. 2013
k. A.	< NWG (0,0006)	Devoy et al. 2013
102	< BG (0,02)	Heitland und Köster 2021
65	< BG (0,003)	Schmied et al. 2021
Außereuropäische Studien		
63 (China)	0,0245 (> NWG)	Liu et al. 2021

^a)^ Morton 2023

^b)^ Wert aus Publikation, aber < BG

BG: Bestimmungsgrenze; k. A.: keine Angabe; NWG: Nachweisgrenze

Vier Arbeiten zur Hintergrundbelastung von Beryllium im Urin in Deutschland liegen vor (Heitland und Köster 2004, 2006 b, 2021; Schmied et al. 2021). Das 95. Perzentil war dabei in allen Fällen < 0,02 µg Beryllium/l. Allerdings waren die 95. Perzentile lediglich bei einer der vier Studien verlässlich quantifizierbar, d. h. lagen oberhalb der Bestimmungsgrenze.

Aus Europa wurden 4 Studien mit Angaben zum 95. Perzentil oberhalb der Bestimmungsgrenze veröffentlicht. Mit Ausnahme der französischen Arbeit von Nisse et al. (2017) liegen die 95. Perzentile dabei zwischen 0,01 und 0,03 µg Beryllium/l Urin. Dies ist in guter Übereinstimmung mit den weiteren acht unterstützenden Arbeiten mit Angaben von Urinkonzentrationen, von denen fünf ein 95. Perzentil von < 0,01 µg/l aufweisen und der Rest mit einem 95. Perzentil zwischen < 0,02 und < 0,05 µg/l vereinbar ist. Eine mögliche Erklärung für die Abweichung der Daten von Nisse et al. (2017) von den restlichen Daten könnte darin liegen, dass die dort untersuchten Personen aus einem früher stark industriell belasteten Gebiet stammten. Anhand dieser Daten und unter besonderer Berücksichtigung der Daten aus dem deutschen Raum wird ein 

**BAR von 0,02 µg Beryllium/l Urin**

abgeleitet. 

## Interpretation

Die Datenlage zur Aufnahme und Ausscheidung von Beryllium zeigt eine Abhängigkeit von der Dosis, Löslichkeit und chemischen Form der Berylliumverbindungen. Die Daten legen jedoch bei chronischen Belastungen keine wesentlichen Einflüsse einer höheren kurzfristigen Exposition nahe. Damit stimmen auch die mehrfach beschriebenen langen Halbwertszeiten in Urin und Blut von bis zu mehr als 100 Tagen überein. Die Probenahme sollte daher erfolgen, wenn sich die Konzentration von Beryllium im Fließgleichgewicht befindet. Aufgrund der langen Halbwertszeit kann es nach (Wieder-)Aufnahme der Tätigkeit längere Zeit (mehrere Wochen) dauern, bis sich ein Fließgleichgewicht einstellt.

Im Hinblick auf kurzzeitige oder einmalige Belastungen, insbesondere über den im beruflichen Kontext vorrangigen inhalativen Aufnahmeweg, ist mit einer um einige Stunden verzögerten Nachweisbarkeit der systemischen Belastung aufgrund notwendiger Resorptionsprozesse zu rechnen. Das Maximum einer Elimination im Urin wäre am ehesten im Bereich von 10 bis 24 Stunden nach Expositionsende zu erwarten (Aviv et al. 2018; Stiefel et al. 1980; Zorn et al. 1986).

Der BAR bezieht sich auf normal konzentrierten Urin, bei dem der Kreatiningehalt im Bereich von 0,3–3 g/l liegen sollte. In der Regel empfiehlt sich bei Urinproben außerhalb der oben genannten Grenzen die Wiederholung der Messung bei normal hydrierten Personen (Bader und Ochsmann 2010).
